# Multi‐disciplinary team approach for pediatric hemimegalencephaly: Insights from a single institutional case series

**DOI:** 10.1002/epi4.13079

**Published:** 2024-10-23

**Authors:** Benjamin Edmonds, Jacqueline P. Ngo, Aran Groves, Beck Reyes, Rolanda A. Gott, Dennis J. Chia, Hilda Mirbaha, Shino Magaki, Negar Khanlou, Stacy L. Pineles, Noriko Salamon, Rachel M. Thompson, Maya Newman, Rajsekar R. Rajaraman, Shaun A. Hussain, Aria Fallah, Bianca Russell, Hiroki Nariai

**Affiliations:** ^1^ Division of Pediatric Neurology, Department of Neurology University of Washington Seattle Washington USA; ^2^ Division of Pediatric Neurology, Department of Pediatrics, David Geffen School of Medicine UCLA Mattel Children's Hospital Los Angeles California USA; ^3^ Division of Developmental Pediatrics, Department of Pediatrics, David Geffen School of Medicine UCLA Mattel Children's Hospital Los Angeles California USA; ^4^ Division of Pediatric Endocrinology, Department of Pediatrics, David Geffen School of Medicine UCLA Mattel Children's Hospital Los Angeles California USA; ^5^ Division of Neuropathology, Department of Pathology and Laboratory Medicine, David Geffen School of Medicine UCLA Medical Center Los Angeles California USA; ^6^ Department of Ophthalmology, David Geffen School of Medicine UCLA Medical Center Los Angeles California USA; ^7^ Department of Radiological Sciences University of California Los Angeles Los Angeles California USA; ^8^ Department of Orthopedics University of California Los Angeles Los Angeles California USA; ^9^ Division of Pediatric Physical Medicine and Rehabilitation University of California Los Angeles Los Angeles California USA; ^10^ Department of Neurosurgery, David Geffen School of Medicine UCLA Medical Center Los Angeles California USA; ^11^ Division of Clinical Genetics, Department of Human Genetics, David Geffen School of Medicine UCLA Mattel Children's Hospital Los Angeles California USA; ^12^ The UCLA Children's Discovery and Innovation Institute Los Angeles California USA

**Keywords:** epilepsy, genetics, hemimegalencephaly (HME), MTOR, multidisciplinary

## Abstract

Recent genetic studies have revealed that hemimegalencephaly (HME) is a multi‐system disorder associated with germline or mosaic variants within the *PI3K‐mTOR‐GATOR1* signaling pathways. Patients with HME typically develop drug‐resistant epilepsy necessitating extensive evaluation, hemispherectomy, and long‐term management. We describe the role of a multidisciplinary team (MDT) for the diagnosis and management of recent patients with HME at UCLA who underwent hemispherectomy. Genetic evaluation identified nine patients with the following variants: *NPRL3* x2 germline, *PIK3CA* mosaicism x4, *MTOR* mosaicism x1, *AKT3* mosaicism x1, unknown x1. Each patient's MDT comprised 4–9 specialties. One child with a *MTOR* variant had persistent epilepsy after hemispherectomy, but addition of everolimus resulted in an 80% decrease in seizure frequency. Another child with hemihypertrophy and *PIK3CA* mosaic variant was offered targeted *PIK3CA* inhibitor treatment, alpelisib, for overgrowth. A third child with germline *NPRL3* variant inherited from their unaffected mother resulted in a sibling being diagnosed with the variant who later developed seizures secondary to focal cortical dysplasia. The implementation of a MDT offers essential guidance for families affected by HME, encompassing prognostication, surveillance, and therapeutic strategies. Identifying the etiology of HME can facilitate the development of targeted treatments and enable timely genetic counseling.

**Plain Language Summary:**

Hemimegalencephaly (HME) is a complex brain disorder caused by genetic changes. It often leads to severe epilepsy that doesn't respond to standard treatments and frequently requires surgery. In this case series, nine patients with HME were identified and found to have genetic mutations in key growth‐regulating genes. A multidisciplinary team model was developed to facilitate patients' care. For example, one patient's seizures improved with surgery, another with a new targeted medication, and another received treatment for symptoms of overgrowth. This team approach provides comprehensive care for patients and can lead to efficient care coordination and implementation of novel therapies.

## 1 INTRODUCTION

Among children with epilepsy, approximately 1–3/1000 harbor hemimegalencephaly (HME). This diagnosis is associated with debilitating refractory epilepsy, often requiring early hemispherectomy,^1^ and coordination of multiple sub‐specialists for comprehensive care. The genesis of HME‐associated epilepsy is multifactorial, including the timing of corticogenesis and the impact of the genetic insult on developing cortical networks.^2^ Sequencing analysis of resected HME specimens has identified pathogenic—and often mosaic—variants in genes associated with HME that are part of the *PI3K‐mTOR‐GATOR1* pathway.^3^

A multidisciplinary team (MDT) approach to complex multi‐system disorders can be effective in optimizing outcomes and improving patient care for specific patient populations.^4^ This has been demonstrated in the management of other neurologic diseases including multiple sclerosis,^5^ neurofibromatosis,^6^ and tuberous sclerosis,^7^ providing a framework for implementing a MDT approach for HME.

Moreover, HME is now being recognized as a multi‐system disease of cellular overgrowth caused by germline or somatic mosaic variants in the *PI3K‐mTOR‐GATOR1* pathway affecting multiple cell lines.^8^ The effect of these somatic mosaicisms is often severe impacting the CNS, integumentary, vascular, musculoskeletal, and endocrine systems.^9^ Therefore, timely diagnosis and coordinated management of symptoms is essential as early hemispherectomy, the current gold‐standard for treatment, offers potential seizure freedom of up to 50%–90%,^1^ and potentially improved developmental outcomes.^10^

The severity of disease and treatment urgency with both surgical and potentially precision therapeutics makes HME an ideal disease process for the implementation of an MDT. Therefore, we proposed a novel MDT approach to HME to facilitate rapid diagnosis, timely genetic testing/counseling, comprehensive care coordination and multi‐specialty management with the potential to offer precision therapies to appropriate patients. We aim to share insights from HME patients who have benefited from our multi‐pronged approach, particularly those who experienced favorable therapeutic outcomes through targeted therapies.

## 2 METHODS

A retrospective study of all pediatric (age <18 years) patients with HME between March 2019 and March 2024 at a single tertiary‐care center was completed. When affected brain tissue was available, samples were tested using established molecular diagnostic panels to identify mosaic variants.^8^ The variant allele fraction (VAF) was noted, and findings were correlated with histopathology, clinical history, and neuroimaging. There were no exclusion criteria.

**FIGURE 1:**
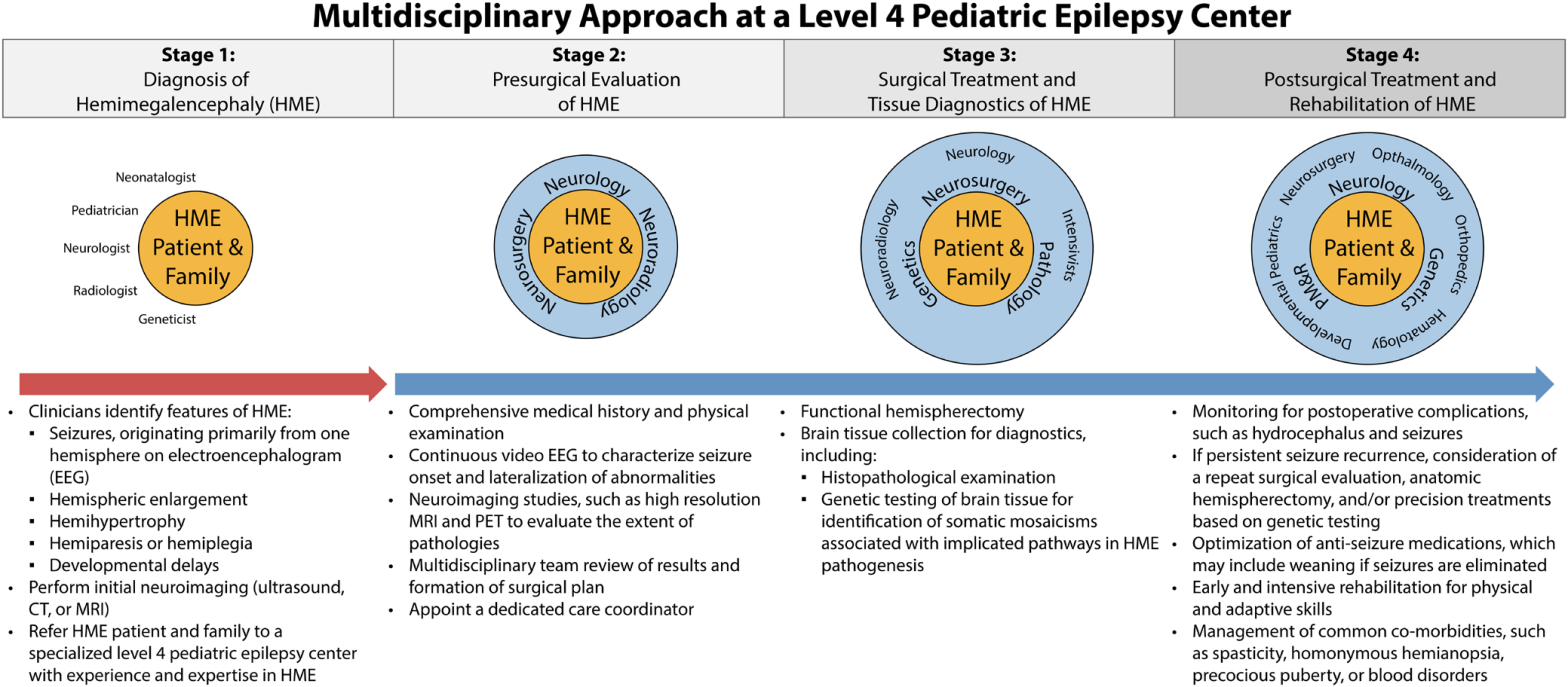
Flow diagram illustrating the steps of our multidisciplinary approach, integrating the appropriate specialties at the diagnostic, presurgical evaluation, surgical, and postsurgical stages.

We identified four main steps of our MDT approach to HME (Figure 1).

### 2.1 Step 1—Diagnosis

Recognition of the disease by primary caretakers and providers is essential. This involves educating neonatologists and pediatricians on key features of the diagnosis, including seizures, subtle asymmetry of the face or extremities, mosaic skin lesions, hemiparesis, developmental delays, and neuroimaging; and ultimately emphasizing the importance of urgent referral to specialists in genetics and neurology at a specialized center.

### 2.2 Step 2—Presurgical evaluation

Establish a “lead” physician to direct care, most often this is the neurologist, given the frequency of communication regarding seizures and medications. In a first consultation with neurology, a pre‐surgical workup is arranged including video‐EEG, neuroimaging studies, and referral for genetic evaluation. Genetic testing with trio exome is completed for all patients without any prior testing. A dedicated care coordinator, at our center this is an epilepsy nurse practitioner, is then appointed to guide the family and help facilitate appointments. The MDT, neurology, neurosurgery, neuroradiology, and genetics, reviews results and determines next steps. Family counseling is critical at this juncture to convey the urgency of treatment, the predicted impact of surgery, and the expected post‐operative journey.

### 2.3 Step 3—Surgical treatment and genetics evaluation

All hemispheric surgeries were conducted by a single surgeon using a modified version of the UCLA Functional Hemispherectomy Technique. This approach is a variant of the lateral peri‐insular hemispherotomy and involves the resection of temporal and peri‐insular structures. The detailed surgical steps for this technique have been previously reported by our team.^11^ Appropriate tissue sampling was coordinated with pathology for both histopathology analysis and targeted low variant mosaic genetic testing if germline testing was unrevealing. This can provide a potential genetic cause for cortical malformation.

### 2.4 Step 4—Postsurgical treatment and rehabilitation

The MDT broadens to provide comprehensive postoperative care and rehabilitation. Developmental and behavioral pediatrics maximizes developmental interventions; ophthalmology follows up on visual disturbances, including hemianopia and strabismus. Orthopedics in collaboration with physical medicine and rehabilitation (PM&R) address acute rehabilitation needs and long‐term sequelae of the resultant spastic hemiplegia/motor dysfunction. Endocrinology monitors precocious puberty, and hematology evaluates blood dyscrasias. In cases of seizure recurrence, repeat surgical evaluation including consideration for anatomical hemispherectomy is warranted. Moreover, genetic testing results are reviewed to determine if targeted therapy can be offered.

## 3 CASE EXAMPLES (TABLE 1)

**TABLE 1:** Multi‐disciplinary results of clinical and genetic data for patients with hemimegalencephaly.

No.	Sex	MRI	Neuropathology	Time of Sz onset	Age at time of surgery (months)	Physical exam findings at diagnosis	Sample type	Genetics	DNA change	Protein change	VAF % Avg	Sz freq/day pre‐surgery	Sz freq (%) improvement post‐surgery	Targeted Tx offered	Sz Freq improvement (%) after Tx	# (specialists)
1	F	L‐HME	FCD type IIa (temporal, perinsular)	Day 1	3	None	Blood	NPRL3 (germline)	Deletion chr16:161898‐1647451x1 (3 kb)	N/a	N/a	20–25	95–100%—less than one clinical sz per day	N/a	N/a	8–Neurology, Nsg, DBP, ortho, PM&R, endo, genetics, ophtho
2	M	R‐HME	Lissencephaly, FCD type IIa (R temporal, peri‐insular, hippocampus, occipital, parietal)	Day 1	2	None	Blood	NPRL3 (germline)	c.274C > T	p.R92T	N/a	Multiple clusters of spasms	80–90%—tonic seizures about once daily	Everolimus	N/a	4–Neurology, Nsg, genetics, hospice
3	F	R‐HME	FCD type IIb (multifocal, posterior temporal lobe)	Day 1	3.5	None	Brain	MTOR (mosaicism)	c.6644C > T	p.S2215F	15%	10–20 focal motor	0%—Still having up to 15 per day	Everolimus	80%—0 to 3 per day	7–Neurology, Nsg, ortho, PM&R, ophtho, GI, genetics
4	M	R‐HME	FCD type IIa (multifocal, temporal, peri‐insular, hippocampus)	6 weeks	4	R cheek hypopigmented	Brain	PIK3CA (mosaicism)	c.1633G > A	p.E545K	13%	3–5 spasm clusters	100%—no further spasms or seizures noted	N/a	N/a	6–Neurology, Nsg, ortho, PM&R, genetics, ophtho
5	M	L‐HME	FCD type IIa (temporal, occipital, parietal, frontal lobes)	Day 1	3	None	Brain	AKT3 (mosaicism)	c.49G > A	p.E17K	5%	20–50 focal motor	100%	N/a	N/a	7–neurology, Nsg, ortho, PM&R, ophtho, endo, surgery
6	F	R‐HME, PMG	FCD type IIa (temporal, peri‐insular, hippocampal regions)	Day 6	4.5	R sided hemihypertrophy, L sided weakness	Buccal swab	PIK3CA (mosaicism)	c.1624G > A	p.E542K	30%	5–10	100%—aside from acute post‐operative seizures, no evidence of seizure recurrence	Alpelisib	N/a	6–neurology, Nsg genetics, ophtho, ortho, PM&R
7	F	L‐HME, PMG	FCD type Ic (peri‐insular regions)	Day 9	56	None	Brain	PIK3CA (mosaicism)	c.3140A > G	p.H1047R	15%	1–2 (per month)	100%	Everolimus	N/a	3–Neurology, Nsg, genetics
8	M	L‐HME	FCD with astrogliopathy (lateral temporal lobe, peri‐insular region)	Day 5	1	Asymmetric ear positioning	Blood	Negative	N/a	N/a	N/a	Innumerable	100%	N/a	N/a	9*–Neurology, Nsg, DBP, CFC, GI, cardiology, genetics, ortho, PM&R
9	M	R‐HME	FCD type IIa (right lateral temporal, right peri‐insular)	3 weeks	2	R sided hemihypertrophy; R chin and neck hyperpigmented macule	Brain	PIK3CA (mosaicism)	c.1636C > A	p.Q546K	25%	>500 spasms	100%	Alpelisib	N/a	4–Neurology, Nsg, genetics, Hem‐Onc

Abbreviations: Avg, average; CFC, craniofacial; DBP, developmental behavioral pediatrics; Endo, endocrinology; FCD, focal cortical dysplasia; F, female; GI, gastroenterology; HME, hemimegalencephaly; Hem‐Onc, hematology/oncology L, left; M, male; Nsg, neurosurgery; Ophtho, ophthalmology; Ortho, orthopedics; PM&R, physician medicine and rehabilitation; PMG, polymicrogyria; R, right; Sz, seizure; Tx, treatment; VAF, variant allele fraction.

* Patient 8 was seen by CFC for asymmetric ear position, GI for failure to thrive, and cardiology for long QT syndrome associated with KCNH2 mutation.

### 3.1 Patient 1

#### 3.1.1 Step 1

Five‐year‐old female was born full term with focal motor seizures noted on day of life (DOL) no. 1 consisting of head extension and rapid eye blinking observed by family and neonatal team. Her clinical seizures ceased after starting levetiracetam, however EEG revealed ongoing subclinical seizures unresponsive to multiple anti‐seizure medications (ASMs). Brain MRI revealed a dysplastic left hemisphere with ventricular enlargement, consistent with HME. Epilepsy gene panel from blood revealed germline deletion in *NPRL3*.

#### 3.1.2 Step 2

At 2 months of age, she was experiencing 90 seizures per day with right hemi‐body tonic–clonic activity arising from the left hemisphere. The MDT reviewed her case and given intractable focal seizures with left HME on MRI recommended a left‐hemisphere functional hemispherectomy.

#### 3.1.3 Step 3

Surgery was performed at 3 months of age with microscopic examination of the resection specimen revealing dyslamination and dysmorphic neurons consistent with FCD type IIa. Post‐operatively, she was seizure free; follow up EEG revealed subclinical seizures from the disconnected left hemisphere, with no spread to the contralateral hemisphere, and no burst suppression or encephalopathic patterns.

#### 3.1.4 Step 4

The patient was weaned to only brivaracetam. At 4‐year follow‐up, she was walking, talking, and toilet‐training. Co‐morbidities include behavioral issues, right‐sided hemianopia, advanced bone age, and right‐sided spastic hemiplegic cerebral palsy. She currently follows with developmental pediatrics, ophthalmology, endocrinology, orthopedic surgery, and PM&R.

Notably, this patient's germline variant was maternally inherited, and mother has history of seizures, unspecified type, that are now controlled, and patient's older brother later developed focal epilepsy due to a focal cortical dysplasia (FCD), radiographically has appearance of FCD type II bottom of sulcus lesion, however no pathology results are available for confirmation as no surgery has been done.

#### 3.1.5 Conclusion

Surgical intervention is the first line intervention for seizures associated with HME. Detection of germline mutations can lead to identification of other at‐risk family members in addition to well‐informed reproductive risk counseling.

### 3.2 Patient 3

#### 3.2.1 Step 1

Four‐year‐old female was born at 35 weeks with onset on DOL no. 1 of frequent multifocal motor seizures and subsequent subclinical seizures identified on EEG (75% right hemisphere, 25% left hemisphere). She was treated with ASMs to the point of burst suppression without improvement. Her MRI at 3 months of age revealed entire right hemispheric overgrowth with hypermyelination, thickened cortex of right temporo‐occipital lobe, asymmetrically enlarged right olfactory tract, and right aberrant fornix consistent with HME, and no associated abnormalities in the left hemisphere.

#### 3.2.2 Step 2

The MDT recommended trio‐exome from blood (which was negative) and pre‐surgical evaluation. An ictal PET showed increased uptake in the right hemisphere with no clear abnormalities on the left. Given recurrent focal seizures and right HME on imaging, the team recommended right‐hemisphere functional hemispherectomy. Development at 3 months was documented to be on track with no signs of regression.

#### 3.2.3 Step 3

At 3.5 months of age, she underwent hemispherectomy. There was an initial post‐operative reduction in seizure frequency; however, recurrent left hemisphere focal motor seizures with rhythmic clenching of right hand were subsequently noted. Microscopic examination of the resection specimen showed multifocal FCD type IIb. Brain tissue sent for detection of low‐level genetic mosaicism revealed a pathogenic mutation in *MTOR* with 15% VAF.

#### 3.2.4 Step 4

At 11 months of age, she was having 20–25 seizures per day originating from the remaining left hemisphere, despite five ASMs and ketogenic diet. A follow‐up brain MRI revealed no signs of ongoing connection in the left hemisphere. Based on her *MTOR* gene mutation supporting the hypothesis of genetic mutation leading to ongoing seizure activity from the seemingly normal left hemisphere, the MDT offered a trial of everolimus, a targeted kinase inhibitor of *MTOR*. Within 1 month, her seizures were reduced to eight per day; with further up‐titration, she had periods of seizure freedom lasting weeks. At a everolimus dose of 5 mg/m^2^ her level has been maintained within the therapeutic range of 5–9 ng/mL. Now at 4 years of age, she only has 0–3 seizures per day on maintenance everolimus, four ASMs and ketogenic diet. Follow up imaging did not show any structural abnormality in the left hemisphere.

#### 3.2.5 Conclusion

Testing for somatic mosaicism allowed for targeted therapy, resulting in a substantial reduction in seizure frequency.

### 3.3 Patient 6

#### 3.3.1 Step 1

20‐month‐old female was born at 37 weeks with notable right hemihypertrophy. On DOL no. 6 she presented with cyanotic episodes, which were classified as seizures on video‐EEG, and she was started on levetiracetam. EEG showed right hemispheric slowing and bilateral epileptiform discharges. Her brain MRI at 10 days old, revealed hypermyelination, overgrowth of right hemisphere and cerebellum, and right hemispheric polymicrogyria: consistent with right HME.

#### 3.3.2 Step 2

At 3 months of age, she developed infantile spasms with hypsarrhythmia on video‐EEG. Vigabatrin was added and her clinical spasms resolved, but follow‐up EEG showed continued right hemi‐hypsarrhythmia and focal right‐onset subclinical seizures. Genetic testing of buccal tissue from overgrown right cheek revealed a pathogenic variant in *PIK3CA* with 30% mosaicism. Ictal PET showed hypermetabolism throughout the right hemisphere. She was also noted at this time to have multiple developmental delays.

#### 3.3.3 Step 3

At 4 months of age, she underwent right functional hemispherectomy. Pathology revealed polymicrogyria, FCD type IIa, and multifocal nodular and marginal glioneuronal heterotopia.

#### 3.3.4 Step 4

Post‐operatively, she developed acute symptomatic seizures that resolved after 4 days. Four weeks postoperatively, she developed symptomatic hydrocephalus, prompting endoscopic third ventriculostomy and shunt placement. At 3 months follow up, she was seizure‐free, reaching, tracking, and babbling, with left‐sided weakness, while continuing levetiracetam and vigabatrin. The MDT recommended treatment with alpelisib^12^ (targeted *PI3Kα*‐selective inhibitor) for treatment of significant extracranial overgrowth, specifically in the buccal and lip region that were concerning for affecting patient's ability to suck and swallow with goal to slow or ideally decrease overgrowth, which was recently started.

#### 3.3.5 Conclusion

Due to multi‐system impact of HME, tissue specific genetic testing was essential in facilitating targeted therapy for hemihypertrophy.

## 4 DISCUSSION

We developed and implemented an effective MDT approach for patients with HME. This review highlights our team‐based methodology for these complex patients as well as currently available treatment options, emphasizing the importance of urgent evaluation, definitive diagnosis and management of multisystem involvement and comorbidities. As such, clinical suspicion for HME should prompt urgent referral to a specialized center to provide efficient and optimal care. Although early hemispherectomy can be curative, as seen in patient 1, there are cases where seizures or systemic issues persist, necessitating additional interventions, as in patients 3 and 6. Furthermore, appropriate tissue collection is critical. The growing recognition of somatic mosaicisms causing overgrowth syndromes, like HME, demonstrates the value in a clinical pipeline for genetic testing that can lead to offering precision therapies.

In this study up to nine specialists were active in the management of each patient in our cohort, to appropriately address these patients' broad needs. The model we created provides patients with comprehensive and longitudinal care from a dedicated lead physician and care coordinator who guide patients from diagnosis to post‐operative recovery and through the potential addition of precision therapeutics and the treatment of associated comorbidities/sequalae.

Finally, while other MDT approaches in neurology have established excellent long‐term monitoring and care coordination models,^6^, ^7^, ^13^ our approach to HME is notable for additionally focusing on rapid genetic evaluation, surgical intervention, and adjuvant precision medicine.

Due to the uncommon nature of this disease, only a small number of patients were reviewed. As such, the breadth of outcomes and interventions reported is limited. We plan to expand the program and integrate additional subspecialities, comprehensive outcome monitoring, and consider utilizing this approach for other surgically remediable genetic epilepsies. However, the evaluation and management of rare disorders, like HME, requires extensive expertise, infrastructure, and other resources. Therefore, working with advocacy and external support groups and securing funding is important to help establish and maintain this model. Additionally, as an extension of the MDT, external support groups have potential to provide a vital resource for families including peer education and emotional support.

In conclusion, we developed an effective MDT approach to HME focused on rapid diagnosis and genetic testing that orchestrates thoughtful care coordination and comprehensive multi‐specialty management with the potential to offer precision therapies for this patient population.

## AUTHOR CONTRIBUTIONS

We certify that all the authors listed made significant contributions to the work and share responsibility and accountability for the results and have approved the final article. B.E. wrote primary draft of the manuscript, preformed literature review, and data acquisition. H.N. provided supervision, study design, and revision of manuscript. B.R., N.S., A.F. helped with study design and manuscript review. H.M., S.M., N.K. helped with pathology review and data acquisition. J.N. and A.G. helped with data acquisition and figure preparation. R.G., R.R., D.C., S.P., S.H., S.M., and R.T. provided manuscript review.

## CONFLICT OF INTEREST STATEMENT

The authors declare no conflicts of interest.

## ETHICS STATEMENT

We confirm that we have read the Journal's position on issues involved in ethical publication and affirm that this report is consistent with those guidelines.

## Data Availability

Protected health information used in this report cannot be disclosed.
